# Euthanasia in advanced dementia: an empirical study of the decision-making process based on advance directives

**DOI:** 10.1093/ageing/afag170

**Published:** 2026-06-22

**Authors:** Djura O Coers, Brigit Ronde, Eefje M Sizoo, Cees M P M Hertogh, Martin Smalbrugge, Carlo J W Leget, Marike E de Boer

**Affiliations:** Medicine for Older People, Amsterdam UMC Locatie VUmc, Amsterdam, Noord-Holland, The Netherlands; Ageing and Later Life, Amsterdam Public Health Research Institute, Amsterdam, Noord-Holland, The Netherlands; Medicine for Older People, Amsterdam UMC Locatie VUmc, Amsterdam, Noord-Holland, The Netherlands; Medicine for Older People, Amsterdam UMC Locatie VUmc, Amsterdam, Noord-Holland, The Netherlands; Ageing and Later Life, Amsterdam Public Health Research Institute, Amsterdam, Noord-Holland, The Netherlands; Medicine for Older People, Amsterdam UMC Locatie VUmc, Amsterdam, Noord-Holland, The Netherlands; Ageing and Later Life, Amsterdam Public Health Research Institute, Amsterdam, Noord-Holland, The Netherlands; Medicine for Older People, Amsterdam UMC Locatie VUmc, Amsterdam, Noord-Holland, The Netherlands; Ageing and Later Life, Amsterdam Public Health Research Institute, Amsterdam, Noord-Holland, The Netherlands; Care Ethics, University for Humanistic Studies, Utrecht, Utrecht, The Netherlands; Medicine for Older People, Amsterdam UMC Locatie VUmc, Amsterdam, Noord-Holland, The Netherlands; Ageing and Later Life, Amsterdam Public Health Research Institute, Amsterdam, Noord-Holland, The Netherlands

**Keywords:** euthanasia, advance euthanasia directive, dementia, decision-making, end-of-life care, qualitative research, older people

## Abstract

**Background:**

In the Netherlands, euthanasia or physician-assisted suicide are criminal offences, yet physicians may be exempt from prosecution if they meet statutory due care criteria. This exemption also applies in situations where a written advance euthanasia directive (AED) is relied upon after decision-making capacity is lost. Although such cases are rare, it remains unclear how physicians, other healthcare professionals and relatives interpret and translate written wishes into ethically and legally defensible practice.

**Methods:**

We conducted a qualitative case study of 14 real-life AED-based euthanasia cases—seven performed and seven forgone—using semi-structured interviews with 21 stakeholders (14 physicians, 10 other healthcare professionals, 5 relatives). Data were analysed with constant-comparison and framework analysis until saturation.

**Results:**

Five interrelated considerations guided each decision-making process: weighing the AED, assessing current wishes, the elusive nature of unbearable suffering, exploring alternatives and putting plans into action. Four contextual factors shaped judgement across cases: the physician–patient relationship, record-keeping, professional experience and seeking validation from others.

**Conclusion:**

Decision-making on AED-based euthanasia is layered and uncertain, requiring physicians to weigh the patient’s earlier written wishes against present expressions, observable suffering and input from others; when these elements cannot be brought together, participants deem euthanasia ethically unjustifiable.

## Key Points

- Five interwoven considerations structure decision-making across cases.
- Fourteen real-life advance euthanasia directive-based euthanasia cases in advanced dementia.
- Stakeholder consensus is important in end-of-life decisions.
- Decisions align past autonomy, present expressions and observable suffering.
- Empirical evidence to guide clinical practice and policy on advance euthanasia directives in dementia.

## Background

In the Netherlands, euthanasia or physician-assisted suicide (EAS) are criminal offences, yet physicians are exempt if they satisfy six statutory due care criteria (Table 1) and report to a Regional Euthanasia Review Committee (RERC).

Article 2.2 of the Termination of Life on Request and Assisted Suicide Act (Wtl) extends this exemption to cases in which a written advance euthanasia directive (AED) replaces current consent once decision-making capacity is lost, provided the criteria apply ‘mutatis mutandis’ (‘not literally, but without misunderstanding’) [1]. Meant to preserve autonomy into late-stage illness, the clause leaves the scope of professional discretion unclear [2, 3]. The criteria presume a communicative relationship with the patient and have been shaped by case law involving competent patients, as well as by guidance from the Royal Dutch Medical Association (RDMA) and the Dutch State Commission on Euthanasia [4–8].

The 2019 District Court ‘coffee-case’ (Appendix S1) and its 2020 Supreme Court confirmation ruled that all due criteria hold under an AED and offered guidance on interpreting criteria when patient communication is minimal or absent [2, 9–16]. This ruling shifted greater interpretive responsibility to physicians, broadening their latitude while leaving ethical and practical dilemmas unresolved [14, 15, 17].

Despite this legal pathway, AED-based EAS remains rare in advanced dementia, with only nine cases reaching the RERC in 2023 and annual numbers fluctuating between 2 and 11 since 2015 [18]. Population ageing and rising dementia prevalence are expected to increase AED numbers and ethical pressure on physicians [19]. Physicians report particular difficulty with three criteria: establishing a voluntary and well-considered request; assessing unbearable suffering; and determining the absence of reasonable alternatives [2, 20–25]. Empirical insight into how these difficulties unfold in real cases remains limited, as most studies used *hypothetical scenarios* [26, 27].

This study examines *actual cases* in which AED-based EAS was performed or forgone, analysing the views of those directly involved: physicians, other healthcare professionals and relatives. The main research question is: ‘How is the decision reached to perform, or to forgo, an AED-based EAS request in real-life cases of advanced dementia?’ Subsidiary questions were: (1) ‘Which challenges do stakeholders encounter during this process?’ and (2) ‘Which factors are weighed throughout the decision-making process?’ The findings aim to inform clinical, ethical and legal debates and provide evidence for guidance on AEDs in advanced dementia.

## Materials and methods

### Design

A qualitative interview case study involving physicians, other healthcare professionals and patients’ relatives was conducted, following the Consolidated Criteria for reporting Qualitative Studies (COREQ) checklist (Appendix S7) [28].

### Participants

Between April and May 2019, a two-part questionnaire was distributed to three physician groups: elderly care physicians and trainees, who care for older people with advanced dementia in home or long-term care settings [26]; ‘Support and Consultation on Euthanasia in the Netherlands’ (SCEN) physicians, who provide assistance and formal consultation on euthanasia, and physicians from the ‘Euthanasia Expertise Centre’ (EEC physicians), who assist in assessment and performance of (complex) euthanasia cases [29].

**Figure 1:**
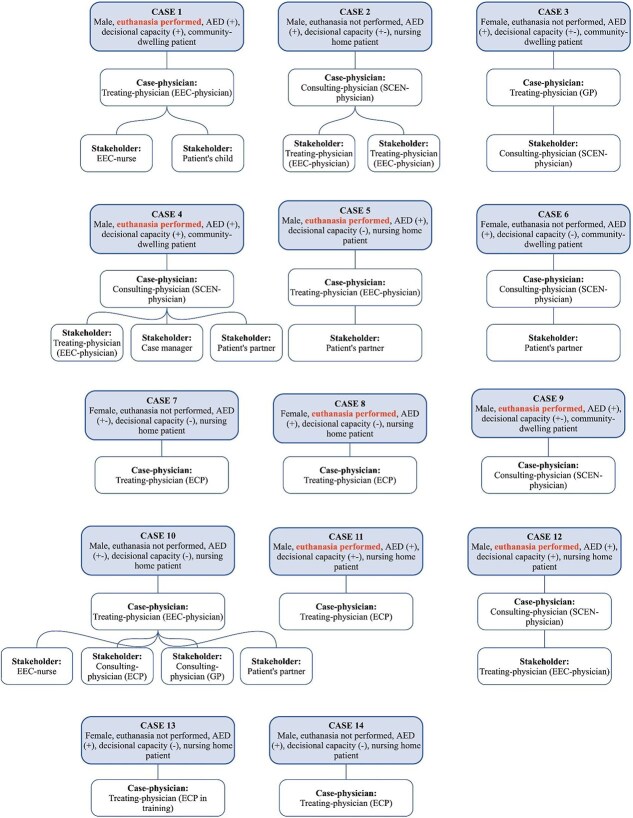
An overview of all cases. Abbreviations: AED: Advance euthanasia directive; EEC: Euthanasia Expertise Centre (EEC physician/nurse; physician or nurse employed by the EEC); SCEN: Support and Consultation on Euthanasia in the Netherlands; GP: General practitioner; ECP: Elderly care physician (in training, where applicable); Case physician: Physician who presented the case; Treating physician: Treating physician with ongoing responsibility for the patient; Consulting-physician: Physician asked to provide an independent opinion or ad-hoc advice (e.g. as SCEN physician) without long-term responsibility; Stakeholder(physician): Physician, other healthcare professional or relative directly involved in the case but not the one who presented it.

Part one explored general views on AED-based EAS (results reported elsewhere [30]); part two asked whether participants had personally handled such a request and, if so, invited them for interview. Purposive maximum-variation sampling (gender, specialty, case outcome) guided interview participant selection. Nineteen eligible physicians were approached; 14 participated (one refusal, four non-responses). Physicians were asked to approach other stakeholders involved in the same case (e.g. physicians, nurses, case managers and relatives) (Fig. 1). Of the 23 stakeholders nominated, 15 participated (two refusal, six non-responses).

### Data collection

Semi-structured interviews (February–July 2020) were conducted face-to-face or via secure video by the first or second author. Stakeholder-specific topic guides (Appendices S2 and S3) covered shared domains—due care criteria, timing, resistance and pre-medication—but differed in emphasis: relatives focused on personal experience and family dynamics; healthcare professionals on team communication, ethics, legal responsibility and organisational aspects. Data collection and analysis proceeded iteratively until thematic saturation. Interviews lasted 45–90 min, were audio-recorded and transcribed verbatim. Field notes documented context and non-verbal cues. Participants reviewed a one-page summary for member checking; eleven returned minor corrections.

### Data analysis

Using a constant-comparison approach [31], two authors (DOC, BR) independently conducted open, focused, axial and selective coding. The code-tree (Appendix S4) was iteratively refined; with consensus on codes and categories reached with another author (EMS). Categories were transferred to a framework-matrix (*MaxQDA*; Appendix S5), to enable constant-comparison within and across cases and systematic thematic analysis [32–34]. Emergent findings were discussed within the research team. Coding and memo-writing used Atlas.ti 7&8 and Microsoft Excel [35]. An audit trail documented codebook versions, analytic decisions and reflexive notes.

**Table 1:** The statutory due care criteria for EAS as stated in the Termination of Life on Request and Assisted Suicide Act^1^

**The physician must**
1. Be satisfied that the patient’s request is voluntary and well-considered.
2. Be satisfied that the patient’s suffering is unbearable, with no prospect of improvement.
3. Have informed the patient about their situation and prognosis.
4. Have come to the conclusion, together with the patient, that there is no reasonable alternative in the patient’s situation.
5. Have consulted at least one other, independent physician, who must see the patient and give a written opinion on whether the due care criteria set out above have been fulfilled.
6. Have exercised due medical care and attention in terminating the patient’s life or assisting in the patient’s suicide.

### Ethical considerations

The Medical Ethics Review Committee of Amsterdam UMC, VU University Medical Centre, approved the study (2019.018). Written informed consent was obtained; transcripts were pseudonymised and stored on encrypted servers.

## Results

### Interviews and participant characteristics

Twenty-nine interviews were conducted. Fourteen physicians presented a case (‘case physicians’): nine with ongoing responsibility for the patient (‘treating-physicians’) and five providing an independent opinion or ad-hoc advice (‘consulting-physicians’). Fifteen additional stakeholders directly involved were interviewed (Fig. 1): seven ‘stakeholder-physicians’ (i.e. physicians who had not presented the case), three other healthcare professionals and five relatives. Figure 1 and Appendix S6 summarise participant and case characteristics.

Although all patients had an AED, its validity was questioned in two cases because it appeared to have been signed after loss of decisional capacity. Euthanasia was performed in seven cases: three patients retained decisional capacity at the time, two had questionable capacity and two had lost capacity entirely.

### Stakeholder perspectives on AED-based EAS cases

Although participants were asked to discuss a single case, many referred to comparable situations or well-known media cases. As these narratives mainly illustrated points already raised, the quotations focus exclusively on the 14 study cases.

**Figure 2:**
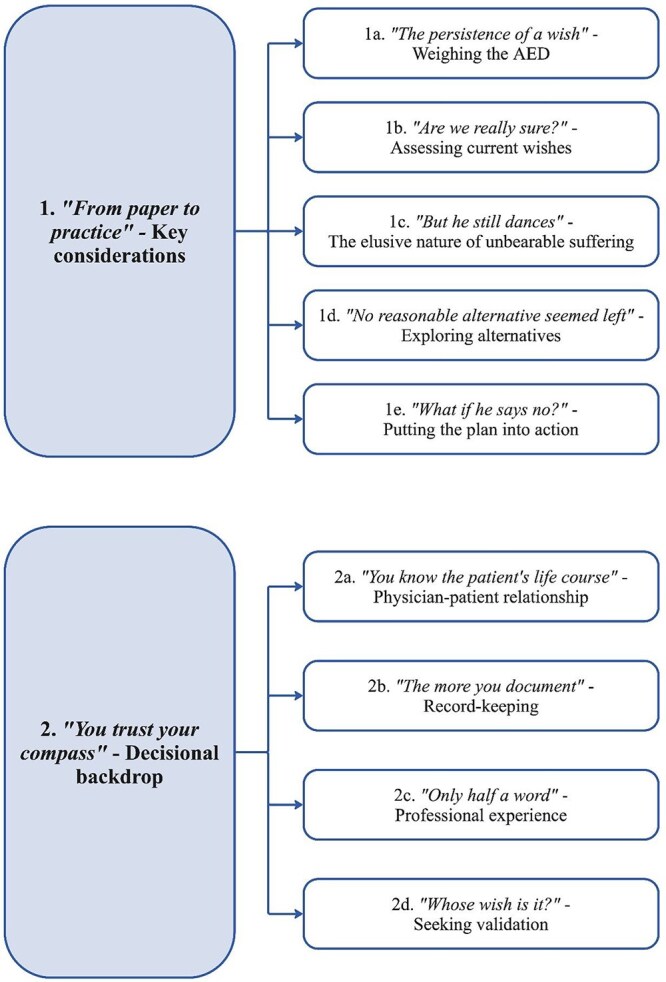
Themes and subthemes.

The presentation of findings follows the research questions. ‘Key considerations’ outlines what physicians, other healthcare professionals and relatives weigh when deciding whether to act on an AED (Fig. 2). ‘Decisional backdrop’ explores four underlying factors shaping decision-making across the process.

Participants also reported the emotional burden associated with decisions about AED-based euthanasia. While highly relevant, this lies beyond the present scope and is mentioned only where it sheds light on decision-making.

#### ‘From paper to practice’—Key considerations

Participants described five interwoven considerations that emerge when AEDs shift from hypothetical reflection to the lived reality of advanced dementia. Terms like ‘struggle’ and ‘dilemma’ were frequent, underscoring the difficulty of these considerations.

##### ‘The persistence of a wish’—Weighing the AED

Three considerations guided AED assessment.

**Value and consistency.** Some physicians treated an AED as a careful yet provisional marker of a will: ‘For me, an advance directive merely confirms the persistence of a wish; it shows the request is well-considered’ (case 4.2, physician; EEC). Others considered the document decisive once it had been discussed consistently over the years: ‘I judged the directive sufficiently clear—it plainly stated she did not want to be in a nursing home, so I regarded it as a voluntary and well-considered request’ (case 8.1, physician; ECP).

**Personal versus generic.** Participants emphasised the value of a personalised AED over a generic form: ‘If people only sign a standard form they have downloaded from the internet, that is their entire directive’ [I encourage to add] ‘a personal story explaining their considerations’ (case 10.4, physician; GP). Another participant noted: ‘It makes a difference when people craft a clear narrative with very specific ideas; personal is better’ (case 3.1, physician; GP). When gaps were identified, physicians tended to involve relatives: ‘If I spot any gaps in the advance directive, I try to fill them in through conversations with relatives’ (case 1.1, physician; EEC).

**Durability.** As a GP explained, a directive’s validity must be reassessed as dementia progresses: ‘That is always the dilemma with dementia patients: they once signed a directive saying they wanted euthanasia in the future, but when that future comes, they are different people with different ideas, so you must check again whether the past opinion still applies’ (case 10.4, physician; GP). This reflects the tension between respecting past autonomy and recognising possible shifts in present preferences.

##### ‘Are we really sure?’—Assessing current wishes

Physicians struggled to assess whether expressed wishes were sufficiently stable. Requests alternated between implicit wishes to die and explicit pleas for euthanasia, while patients often lacked sustained comprehension: ‘She sometimes said “just give me that injection”, but when I explained that the doctor would give a shot and she would die immediately, she could not hold on to that idea’ (case 3.2, physician; SCEN). When decision-making capacity was ‘tested with the Appelbaum criteria’ [36], ‘the level of reflection he was trying to elicit was simply gone’, a physician noted; ‘but he could very clearly write “dead”, “gone” on scraps of paper all day’ (case 10.1, physician; EEC). The absence of contemporaneous verbal confirmation raised doubt: ‘It makes an enormous difference when a patient can still say, “Doctor, go ahead”. If not, you ask yourself, “Are we really sure?”’ (case 8.1, physician; ECP). Even with a directive in place, ‘they still lack the possibility to say at the last moment “yes, I want it” or “no, I don’t”. That is a very, very important element for me’ (case 14.1, physician; ECP). Aligning conflicting utterances was not a checklist exercise: ‘If he once says “not after all” but the nurses hear him say “I want to” twenty times a day, I still need to hear it again from him; the overall picture must be persuasive, it is not a checklist’ (case 1.1, physician; EEC).

##### ‘But he still dances’—The elusive nature of unbearable suffering

The elusive nature of unbearable suffering was shaped by two key considerations.

**Ambiguous signals of suffering.** Efforts to identify the source of distress sometimes yielded little: ‘When you ask “why not”, “what is the worst for you?” or “what do you suffer from?”, nothing surfaced; she couldn’t put it into words’ (case 7.1, physician; ECP). Yet healthcare professionals in the same case observed severe daily anguish: ‘From roughly noon until five in the morning she cried, appeared frustrated, and was very angry’ (case 7.1, physician; ECP). Physicians emphasised that observable signs were decisive: ‘If a patient now sits contentedly on the couch, that visible absence of suffering means the unbearable-suffering criterion is not met’ (case 1.1, physician; EEC). Another participant stressed the need for direct observation: ‘The suffering has to be the person’s own—and I must be able to see it. Not just rely on a compelling story or an advance euthanasia directive. I need to truly see that, for these people, this life is absolutely no longer a life worth living’ (case 10.2, other healthcare professional; nurse). A physician described the demands of this assessment: ‘Forces me to be meticulous, go deeper, ask more, lay the patient’s soul bare, until I feel convinced that the request truly fits the legal framework and is genuinely the patient’s wish… As the performing physician, the suffering has to be palpable to me’ (case 4.2, physician; EEC). By contrast, pronounced non-verbal distress—‘saying “no, no, no”, kicking, hitting, agitation, crying’ (case 1.1, physician; EEC)—could provide visible evidence to establish unbearable suffering. Yet doubt could persist despite repeated assessments: ‘On a second visit I was still not convinced there was unbearable suffering; he needed a lot of help and could communicate only intermittently’ (case 2.2, physician; EEC).

**Projection by relatives.** Physicians often found it harder to substantiate suffering than relatives: ‘We explained that what you label beforehand as unbearable no longer counts; we must assess current, actual suffering’ (case 10.1, physician; EEC). A participant described how distress may shift from patient to family: ‘The suffering shifts—from the patient to the family. Then it is no longer his suffering but theirs. We saw that shift very clearly with this young man, and it became very difficult to disentangle his unbearable suffering from that of those around him’ (case 10.2, other healthcare professional; nurse). Relatives’ own burden or difficulty accepting change sometimes coloured their judgement: ‘In my view it was more about an overburdened home situation and a wife who could not accept her husband’s changes’ (case 6.1, physician; SCEN). A spouse—still upset with the ECP who had rejected the request—recalled the remark ‘But he still dances’ (case 5.2, relative; spouse), illustrating the gap between professional and family perspectives. While the physician viewed the retained ability to dance as a sign of acceptable quality of life, the spouse described her husband’s daily ordeal—recurrent falls with bruising, catheter-managed incontinence and nights of anguished shouting—as ‘suffering you wouldn’t wish on a dog’ (case 5.2, relative; spouse). Another relative recalled: ‘In the end the request was rejected because the physicians could not clearly see that he was suffering—let alone unbearably—and thought the suffering lay more with us than with him… It hit us hard when the request was denied’ (case 10.5, relative; spouse).

##### ‘No reasonable alternative seemed left’—Exploring alternatives

Several participants questioned whether patients with advanced dementia could still assess and compare alternatives: ‘You must be able to see alternatives and evaluate them, and he could do that only to a very limited extent’ (case 2.2, physician; EEC). Three main alternatives were considered.

**Nursing-home admission.** Physicians often considered relocation, noting some patients eventually thrived: ‘In my nursing-home years I saw people who dreaded admission but blossomed after a few months’ (case 3.2, physician; SCEN). Explicit refusals in AEDs could preclude this option, placing physicians in a dilemma. Even when admission proceeded, outcomes could disappoint: one patient who initially opposed the idea agreed once home care failed but ‘hoped he would function better among people; that absolutely wasn’t the case—it became very unpleasant for him’ (case 12.1, physician; SCEN).

**Targeted psychopharmacology for behavioural symptoms.** When severe agitation or aggression predominated, physicians sometimes used short-term neuroleptics alongside behavioural interventions: ‘We once had someone terribly restless, unhappy, aggressive; a short course of neuroleptics calmed him until the dementia progressed and he seemed to enjoy life again’ (case 9.1, physician; SCEN). In other cases, all options were exhausted without success: ‘The specialist felt we had tried everything, pharmacologically and behaviorally, and saw no further options’ (case 8.1, physician; ECP).

**Palliative pathway: continuous deep palliative sedation.** When the due care criteria were not met, some physicians opted for a comfort-focused alternative: withdrawing life-prolonging interventions and, when appropriate given limited remaining life expectancy, initiating continuous deep palliative sedation. For relatives, this slower dying process could be difficult to bear: a relative recalled that this course ‘took a long time… the dying process itself… I feel guilty that his suffering lasted longer than we wanted because euthanasia attempts failed’ (case 10.5, relative; spouse).

##### ‘What if he says no?’—Putting the plan into action

Three interlinked elements shaped challenges in executing AED-based EAS.

**Humane performance.** Many participants described short-acting pre-medication (e.g. low-dose oral midazolam, distinct from continuous palliative sedation) as enabling a humane procedure and avoiding coercion: ‘If he resisted we had agreed with the family in advance that we would stop immediately; we are not going to hold someone down’ (case 4.2, physician; EEC). Once legal requirements were met, ‘it is more responsible to mix the premedication in his coffee than to restrain him and inject it’ (case 1.1, physician; EEC), a physician argued. Another acknowledged the personal cost: ‘It means I have to swallow hard as a physician, because I really don’t like doing it’ (case 10.1, physician; EEC). Relatives also endorsed the use of pre-medication—‘That Dormicum [i.e. Midazolam]… no problem, it had to proceed calmly’ (case 5.2, relative; spouse).

**Moral friction and uncertainty.** Despite careful preparation, doubt could persist: ‘The night before I slept maybe an hour—what if he says no at that moment?’ (case 4.3, other healthcare professional; case manager). Uncertainty was compounded by practical gaps, notably the lack of formal guidance on oral midazolam dosing when IV access was not feasible: ‘I consulted the pharmacy and several physicians and still did not know what dose to use; we had to improvise’ (case 8.1, physician; ECP).

**Planning ahead.** To manage moral friction, teams used planning as a coping strategy: ‘We then made a plan for how we would carry it out, including sedation beforehand. We talked through the day, drafted a script for it. We already had one, because I had performed euthanasia twice before on this ward, two years earlier, so we could fall back on that. On the scheduled day we carried out the euthanasia according to plan’ (case 8.1, physician; ECP). A clear plan also reassured relatives: ‘Everything was very clear’, the patient’s daughter confirmed (case 1.3, relative; daughter). Although legal accountability rests with the performing physician, participants experienced planning and execution as shared team responsibility, helping distribute tasks and ease the emotional burden.

#### ‘You trust your compass’—Decisional backdrop

This decisional backdrop comprises four interrelated subthemes describing contextual factors shaping decision-making throughout the AED-based EAS. Unlike formal guides—such as the legal due care criteria or the AED itself—these factors operated more subtly, influencing how formal requirements were interpreted.

##### ‘You know the patient’s life course’—Physician–patient relationship

Physicians emphasised that long-standing relationships provide crucial insight into patient’s values and life trajectories: ‘The advantage is you know the patient’s life course… you can better judge whether euthanasia fits their worldview’ (case 10.4, physician; GP). Another GP noted that a years-long bond ‘makes it both harder and easier, because you know exactly what someone wants’ (case 6.1, physician; SCEN). Ongoing documentation further strengthened decisional confidence: ‘When I saw her suffering, I knew we had talked everything through and recorded it over time; then I could proceed’ (case 3.1, physician; GP).

##### ‘The more you document’—Record-keeping

Thorough documentation, expert reports and timely exchange of medical records gave physicians firmer footing for decision-making. As a GP observed: ‘You know that the more you document and involve another expert—an old-age psychiatrist, a geriatrician, whoever—and have them produce a professional report on competence and suffering, it makes a great deal of difference’ (case 3.1, physician; GP). Another participant highlighted the value of detailed advance care planning: ‘He even drew up a medical living will and had it notarized. He hoped, of course, that his wishes would be honored, and he really covered every base. For us, that meant it wasn’t much of a hurdle to take the next step and help him’ (case 1.2, other healthcare professional; nurse). Consulting physicians typically first checked dossier completeness, requesting missing information as needed: ‘Could you send us the relevant pages from your record, an episode overview, and any specialist letters?’ (case 1.1, physician; EEC). A comprehensive dossier enabled timely assessment and signalled when additional expertise was required.

##### ‘Only half a word’—Professional experience

Experienced EAS physicians explained that their first step was to ‘call the GP and ask why they could not perform the euthanasia themselves—often it is because euthanasia in dementia is too complex and they prefer someone with more experience’ (case 1.1, physician; EEC). Another physician noted that limited time, expertise and practical tools could preclude GPs from performing the procedure: ‘The GP was closely involved in the execution but simply lacked the tools to do this complicated work’ (case 4.1, physician; SCEN). Reflecting on early experiences, a nurse recalled: ‘We [the nurse and the EEC-physician] were, of course, still very much finding our way through all of this. We didn’t have much experience yet, and we didn’t know each other all that well either’ (case 10.2, other healthcare professional; nurse). After many complex cases, coordination now required ‘only half a word’ to manage the ‘choreography’ of the decision-making process (case 10.2, other healthcare professional; nurse). Repeated experience also fostered physicians’ confidence: ‘That experience makes me strong… if I doubt, it is with good reason’ (case 12.2, physician; EEC). Families recognised this expertise: ‘They treated my father with great respect and asked thorough questions… I felt they had experience with people with dementia’ (case 1.3, relative; daughter).

##### ‘Whose wish is it?’—Seeking validation

This theme examines how participants seek external confirmation that the request originates with the patient and meets the due care criteria.

**Conflicting voices.** Assertive relatives sometimes raised doubts about whose wish was being expressed: ‘In my conversation it felt more like the partner’s wish than the patient’s. He merely echoed what his partner said, so I concluded the due care criteria were not met’ (case 6.1, physician; SCEN).

**Confirming the patient’s wish.** When patients could no longer fully express themselves, physicians sought external confirmation: ‘She told the nursing staff every day that she wanted to die, and her daughter even made a recording in which the patient clearly said she wanted to die—an important supporting document’ (case 3.2, physician; SCEN).

**Professional cross-checks.** When a tentative decision emerged, physicians often sought peer validation of their overall judgement: ‘Well, it was important to me to know what all the other parties thought. You check with each person—“what does this one think, what does that one think?”. Then you simply line everything up and, of course, follow your own gut. That way you feel more confident in your gut feeling’ (case 10.3, physician; ECP). Another participant highlighted the value of informal collegial sparring: ‘I got a lot out of my own colleagues—just bouncing ideas off each other, you know. This isn’t everyday work for us, so it’s really helpful to have two other colleagues you can talk with about what you’re seeing, what you’re noticing, what they would do’ (case 4.3, other healthcare professional; case manager).

**Avoiding tunnel vision.** Physicians acknowledged the risk of a single narrative. One participant admitted she had ‘been sucked into the spouse’s perspective’ until a consultation ‘held up a mirror’, enabling her to regain distance and decide not to proceed (case 6.1, physician; SCEN).

## Discussion

### Main findings

This case study is the first empirical analysis of both performed and forgone AED-based EAS in advanced dementia. Five key considerations emerged: weighing the AED; assessing current wishes; evaluating unbearable suffering; exploring alternatives; and putting the plan into action. In addition, four decisional backdrop factors shaped decision-making: physician–patient relationship; record-keeping; professional experience; and seeking validation. Despite the Dutch Supreme Court’s ruling, substantial uncertainty remains. Our findings show how stakeholders navigate the path from an AED to a morally defensible outcome within the legal framework. Although the final decision formally rests with the physician, all stakeholders emphasised the need for shared consensus. Whether euthanasia is performed or forgone, decision-making requires continuous calibration of past autonomy against present expressions, observable suffering and differing stakeholder perspectives.

### Interpretation of the findings

For readability, the subsections are organised around themes that broadly, though not strictly, parallel the due care criteria.

#### Voluntary and well-considered request

Our finding that healthcare professionals closely examine AED consistency and personalisation aligns with a recent Delphi study showing that generic, one-page forms are insufficient and that current behaviour and expressions should override an AED, even after loss of decisional capacity [3]. Accordingly, physicians tended to regard an AED as ‘decisive’ when the wish had been repeatedly revisited and supported by documentation from multiple observers, but were reluctant when current expressions or behaviour contradicted it. Participants repeatedly weighed the AED against the patient’s current expressions, behaviour and broader clinical context. When current expressions or behaviour diverged from the AED, tension could arise between respecting precedent autonomy and what the ethical literature calls ‘upholding the dignity of the present person’ [36]. Such findings are consistent with calls for interpretive dialogue and attention to the dignity of the present person rather than treating the AED as automatically decisive [36].

#### Decisional capacity and current wishes

A qualitative RERC review of 60 dementia-related EAS cases found that physicians rarely applied formal capacity criteria, judging capacity mainly by a patient’s ability to engage in coherent two-way communication, with thresholds shaped by communication level, request consistency, AED presence and the patient–physician relationship [37]. Our interviews suggest that once such cues fade, professionals rely on converging reports from nurses and relatives, echoing earlier research describing a consensual model in which physicians actively seek input from the care team when direct dialogue is no longer possible [2]. Although our study did not examine assisted decision-making legislation directly, these findings resonate with broader ethical and legal debates, including those under the UN Convention on the Rights of Persons with Disabilities [38], emphasising autonomy through relational and interpretive decision-making rather than substituted judgement alone [36, 39]. In our cases, the AED was rarely treated as self-sufficient; instead, participants described an interpretative process in which earlier wishes were weighed against current expressions, behavioural cues and input from relatives and professionals. Rather than replacing the patient’s voice, this process aimed at reconstructing, as carefully as possible, whether the AED still cohered with current expressions and apparent wishes. Nevertheless, physicians remain uneasy without a final patient ‘yes’, captured in the recurring question ‘Are we really sure?’, underscoring a gap between legal permissibility and bedside moral certainty in AED-based euthanasia.

#### Unbearable suffering

Traditionally, unbearable suffering is a subjective, patient-centred criterion requiring physician conviction and presupposing dialogue with the patient. The Supreme Court’s 2020 ‘coffee-case’ ruling held that, in the absence of a patient’s contemporaneous articulation of suffering, physicians bear responsibility for judging whether the suffering can be considered unbearable under the due care criteria [40–42]. In those cases of advanced dementia where dialogue is no longer possible, assessments shift towards observable behaviour and converging reports from caregivers and relatives. Consistent with earlier research showing this to be the most challenging criterion [30], non-verbal distress became the primary indicator, while relatives often perceived suffering earlier. The physician’s remark ‘But he still dances’ illustrates the interpretative gap between physicians and relatives, widely recognised as a point of dispute regarding unbearableness [17]. These tensions appeared mainly as a discrepancy between professional and family interpretations of suffering, especially when family members perceived suffering earlier or more intensely than physicians. However, previous literature suggests that family members may also differ among themselves in how they interpret the patient’s situation and prior wishes [43, 44]. Such differing perspectives may complicate decision-making while helping prevent overly narrow or one-sided interpretations of suffering. Other studies note the absence of a shared definition of ‘unbearable suffering’ and propose conceptualisations framing it as a deeply personal, multifaceted experience, encouraging inclusion of both patient and family perspectives [45].

#### Alternatives

The ‘coffee-case’ confirmed that all due care criteria—including the absence of *reasonable alternatives*—remain applicable after loss of decisional capacity, with full responsibility resting with the physician [40–42]. Although patients with advanced dementia are not required to weigh alternatives themselves, several participants described unease when assessing what counts as a ‘reasonable’ alternative without the patient’s input: ‘You must be able to see alternatives and evaluate them, and he could do that only to a very limited extent’. This self-imposed requirement reflects a shared decision-making ethos, despite no longer being legally mandated. It also highlights a normative tension: determining the reasonableness of alternatives involves value judgements ordinarily made by the patient, yet now inferred by physicians without dialogue. The assessment of alternatives should be framed within the broader palliative care continuum, where options are typically explored and family members play a central role. While Dutch law recognises relatives as key discussion partners once decisional capacity declines [44], in AED-based euthanasia their formal role is largely limited to conveying prior wishes [46, 47]. Physicians should therefore clarify this distinction to align expectations, reduce conflict and strengthen the transparency and ethical defensibility of the ‘no reasonable alternative’ assessment.

#### Execution practice

On the day of the procedure, participants balanced moral unease with careful coordination. Many favoured a small pre-procedural dose of sedative medication because, as one physician stated: ‘It is more humane to slip Dormicum [i.e. Midazolam] in his coffee than to hold him down’; a calm patient was considered more dignified than one who struggled. Although concealed sedation has been considered permissible within the Dutch legal framework for EAS in advanced dementia, ethical debate persists and guidance on timing and dosage remains limited [48, 49]. This practice should be distinguished from continuous deep palliative sedation, which is aimed at refractory symptom relief in the context of a limited life expectancy [48, 49]. In our cases, pre-euthanasia sedation was described as a brief, low-dose measure intended to facilitate a calm and non-coercive euthanasia procedure. Without clear guidance on the use, timing and dosage of pre-euthanasia sedation, proceeding without an immediate ‘yes’ remained troubling, but detailed planning with the team and family helped mitigate this tension. Pre-euthanasia sedation and its associated moral unease receive limited attention in the literature; to our knowledge, no empirical studies have systematically examined this practice in advanced dementia. By documenting current use of pre-procedural sedation, this study adds nuance to the post-2020 Supreme Court debate. Participants framed low-dose midazolam as a pragmatic means of keeping procedures calm and non-coercive, yet the lack of formal guidance continues to generate uncertainty and warrants further normative analysis. This ethical unease stems not only from uncertainty about dosing or timing, but also from doubts about whether sedation acceptably facilitates a calm, non-coercive procedure. International comparison is limited, as AED-based euthanasia in advanced dementia is exceptional to the Dutch context [36]. This underlines the need for clearer national guidance and further ethical analysis of the circumstances under which pre-euthanasia sedation may be considered acceptable.

#### Decisional backdrop

Although AED-based euthanasia remains rare, repeated involvement appeared to foster procedural confidence; as one physician noted, coordination sometimes required ‘only half a word’ from colleagues. Nevertheless, moral dilemmas—particularly before execution—persisted, as even experienced physicians continued to weigh others’ views before judging whether the due care criteria were met. Our findings suggest that emotional burden may influence clinical decision-making in AED-based EAS trajectories. Participants described persistent doubt, sleeplessness before the procedure and the need for detailed planning and collegial coordination, indicating that emotional strain importantly shaped how these trajectories were experienced and managed. In addition, relatives’ own distress and expectations could complicate communication and further challenge the assessment of suffering [20]. This points to the importance of emotional support, in addition to procedural support, for healthcare professionals and relatives. This persistent uncertainty echoes calls for a consensual approach in which physicians actively seek and balance input from relatives and other healthcare professionals when direct patient dialogue is impossible [2]. A recent Dutch survey reports routine consultation of colleagues, SCEN physicians and family members, alongside broad support for more formalised multidisciplinary deliberation [20]. Record-keeping proved pivotal: thorough documentation and expert reports strengthened the medical file and signalled when multidisciplinary meetings were needed. Legal experts identify comprehensive documentation as a concrete—and relatively easy-to-strengthen—element of AED-based EAS care pathways [17].

### Implications for practice and policy

First, our findings suggest that generic one-page AEDs, combined with declining decisional ability, leave physicians with persisting uncertainty. Earlier and more substantive advance care planning—focused on personalising the AED and building a well-documented dossier from multiple observers—may reduce this uncertainty. Second, national guidance on indications and dosing of sedative medication, with clear recommendations on team choreography on the day of the procedure, could alleviate physicians’ moral friction. Although legal accountability rests with the physician, our data indicate that AED-based euthanasia typically unfolds as a collective endeavour; clarifying roles may distribute responsibilities and ease the emotional burden. Third, we propose extending the KNMG requirement for independent SCEN consultation by introducing structured multidisciplinary case reviews before and after each AED-based EAS process [50]. Such debriefings may help teams ‘avoid tunnel vision’ and reduce emotional strain. Although rooted in the Dutch legal context, our findings may also inform contexts beyond the Netherlands. For jurisdictions considering regulation of assisted-dying practices in dementia [51], our study highlights practical and ethical pressure points requiring guidance: interpretation of prior wishes, the role of current expressions, relative involvement, multidisciplinary deliberation and conduct of the procedure.

### Strengths and limitations

A key strength is the real-life scope: analysing both performed and forgone AED-based EAS cases and interviewing stakeholders revealed nuances and gaps between legal frameworks and everyday practice not captured previously. Triangulation across physicians, nurses/case managers and relatives exposed discrepancies a single-source design would miss, though some themes were discussed only by physicians. Long-standing relationships between interviewees and patients added temporal depth and insights into requests and their context.

The study also has limitations. Selection bias cannot be excluded, as physicians determined which stakeholders were approached, potentially over-representing healthcare professionals and relatives with clearer or difficult experiences. In several cases, consulting rather than treating physicians participated, potentially shaping the narratives. The limited number of non-physician professionals narrowed those perspectives, and dual roles (e.g. EEC physicians or SCEN physicians) may have influenced how feasibility and expertise were framed. Because several physicians were affiliated with the EEC or had SCEN experience, our sample may overrepresent physicians with high exposure to, and possibly greater openness towards, ethically complex euthanasia requests. This may have influenced how much interpretive room within the due care criteria participants perceived, and how conceivable AED-based euthanasia appeared in practice. Dutch research suggests that attitudes towards euthanasia in advanced dementia vary substantially between physicians and the public [52, 53], so our findings should not be taken as representative of physicians generally. This limited our insight into interdisciplinary dynamics, particularly how nurses, case managers and other professionals experienced and shaped the interpretation of suffering, team deliberation and the enactment of AED-based EAS. Consequently, multidisciplinary decision-making may be represented more from a physician perspective than the broader care teams. Relative interviews often followed emotionally charged events and, despite reflexive techniques, were likely shaped by grief. Moreover, the findings are embedded in a Dutch legal-cultural context and should be transferred cautiously to other jurisdictions. The Dutch procedural pathway for AED-based euthanasia in advanced dementia is unusual; the legal scope, physicians roles and cultural context may differ substantially elsewhere [51]. Finally, the retrospective design relied on recall rather than observation; prospective, real-time documentation—though ethically complex—may reveal additional nuances. Despite these limitations, the study offers novel insights into AED-based EAS decision-making in advanced dementia.

## Conclusion

Decision-making on AED-based EAS in advanced dementia is a layered and uncertain process, requiring alignment between past autonomy, present expressions and broad consensus supporting the physician’s judgement within a legal framework that—despite Supreme Court ruling—continues to leave room for doubt in daily practice. Without such alignment, participants deem euthanasia ethically unjustifiable. Richer advance-care conversations, national guidance on pre-procedural sedation and execution protocols and regular multidisciplinary reflection may help physicians navigate persistent moral friction, even in highly experienced teams.

## Supplementary Material

Supplementary_materials_afag170
