# Supplementary material for: Euthanasia in advanced dementia: an empirical study of the decision-making process based on advance directives
Source: Age Ageing. 2026 Jun 22;55(6):afag170. doi: 10.1093/ageing/afag170 (PMC13284701; doi:10.1093/ageing/afag170)

**Euthanasia in advanced dementia:** an empirical study of the decision-making process based on advance directives.

**APPENDICES**

**Table of Contents**

| **Appendix** | **Title** | **Page** |
| --- | --- | --- |
| Appendix 1 | Case description of the landmark Supreme Court ruling (the “coffee-case”) in 2020 | 2-4 |
| Appendix 2 | Topic Guide Interview with Family Members | 5-6 |
| Appendix 3 | Topic Guide Interview with Healthcare Professionals | 7-9 |
| Appendix 4 | Code tree – Euthanasia in advanced dementia | 10-12 |
| Appendix 5 | Framework matrix | 13 |
| Appendix 6 | Participants' characteristics and roles in the EAS process | 14-18 |
| Appendix 7 | COREQ checklist | 19-20 |

**Appendix 1 |** Case description of the landmark Supreme Court ruling (the “coffee-case”) in 2020.

| In 2008, the patient was diagnosed with Alzheimer’s disease. Four years later, in 2012, she formulated an AED outlining her preferences to avoid institutionalized dementia care and to pursue voluntary EAS when she would reach a state of cognitive impairment that hinders her ability to provide informed consent for medical decisions preventing her from living at home. In 2015, she updated her AED to include the request for EAS whenever she deems the time is right due to a significant decline in the quality of life. Her general practitioner and gerontologist independently verified her competency at that time^48^.  As her cognitive decline progressed, she was eventually admitted to a nursing home in March 2016. At this stage, her husband requested EAS based on the AED. The ECP conducted a thorough investigation, involving observations of the patient, limited conversations with her due to the advanced stage of dementia, discussions with her relatives, former GP, psychologist, an expert from the Euthanasia Expertise Center and other healthcare professionals.  Despite displaying calm behavior on a daily basis, the patient also exhibited signs of severe restlessness and unhappiness. She expressed her desire to die regularly to the nursing staff but also conveyed to her ECP several times that she did not yet want to die because her suffering had not reached an unbearable level. Based on the ECP’s investigation and the AED, the ECP determined that the patient was no longer capable of making EAS decisions but concluded that EAS was permissible in accordance with the AED. Two independent physicians subsequently examined the case and confirmed that all due care criteria were met.  In April 2016, the ECP performed the EAS, and beforehand, a sedative was administered to the patient’s coffee to prevent agitation, as agreed upon with the family. However, during the administration of the lethal medication, the patient attempted to sit up, requiring her family to hold her down to complete the procedure.  Key point of the legal consequences of the case:  The ECP reported the case to the RERC, as mandated by law. The committee determined that the ECP had not fulfilled the statutory due care criteria of a voluntary and well-considered request and the exercise of due medical care^48^. Consequently, the case was referred to the Health and Youth Care Inspectorate and the Public Prosecution Service, leading to disciplinary and criminal proceedings.  Firstly, the Regional and Central Disciplinary Court opined that the ECP should not have complied with the EAS-request based on the AED^9,10^. The court cited the lack of unambiguous content in the AED and the inconsistency of the patient’s expressions regarding her euthanasia request. Additionally, the court deemed it essential for the ECP to have discussed the patient’s intention to end her life right before administering the lethal medication. As a result, the Regional Disciplinary Court reprimanded the ECP, although the Central Disciplinary Court later reduced the reprimand to a warning, concluding that the ECP’s actions were reprehensible to a limited extent.  Secondly, the District Court acquitted the ECP of all criminal charges, stating that verbal verification of the patient’s current life-or-death wish was not necessary due to the patient’s advanced stage of dementia and specific position as an individual who lacks the ability to make informed decisions about her medical care and other important matters due to her cognitive impairment^11^. The court argued that demanding such verification would undermine the purpose of the AED, which is intended for situations where individuals write an AED and later find themselves in a state of unbearable suffering with no prospects of improvement and are unable to express their desires.  In 2020, the Supreme Court upheld the judgment of the District Court and dismissed the decision of the Disciplinary Court^12,13^. The Supreme Court emphasized that interpreting the EAS-request should not be limited to the exact writing of the AED but should also consider other circumstances, including the patient’s expressions and the beliefs of relatives and other involved healthcare professionals. Furthermore, the Supreme Court clarified that the administration of sedative medication beforehand does not contradict the requirement of due medical care, as EAS should be performed in the most comfortable manner possible for the patient. |
| --- |

**Appendix 2 |** Topic Guide Interview with Family Members*

| **Topics** | **Sub-topics** | **Example questions** |
| --- | --- | --- |
| Case / situation outline | Participants’ background information | - What is the participant’s relationship to the patient? - How would you describe the participant’s bond with the patient? - What is the participant’s role in relation to the euthanasia request? |
|  | Patients’ background information | - Who is or was the patient? (age/course of illness/work history/life philosophy or religion) - What is the patient’s current situation? |
| Euthanasia process and (possible) performance | Background to the wish for euthanasia and the written advance euthanasia directive | - When was the wish for euthanasia first discussed? What prompted this? - When was the written advance directive drawn up? What was the reason for doing so? - What did the directive look like? What did it contain? - What did you think of the patient’s wish for euthanasia / the content of the directive? - Were you able to support this wish / the directive? - How did the patient come to this wish for euthanasia / to drawing up the euthanasia directive? - Which family members or loved ones were involved in the wish / directive? - What do you think of your relative’s wish for euthanasia? - Which healthcare professionals (physician, case manager, nurse) were involved in the wish / directive? - From whom and where did the patient obtain information about this subject? - Did the wish for euthanasia change over time / was the written advance directive revised? |
|  | Current request based on the written advance euthanasia directive | - What situation had arisen when the current request for euthanasia was raised? - What was the reason for the current request? Who made the request? - With whom did the participant discuss the current request (other family members, physician, case manager, etc.)? How did the physician respond to the request? - Did you find it difficult to discuss the euthanasia request with the physician? - Did you feel heard by the physician when euthanasia was discussed? |
|  | Voluntary and well-considered | - Do you think the patient would still have stood behind the directive? What makes you say that? - During the current request, did you try to discuss this with the patient (despite decisional incapacity)? - Do you think a written directive can replace a verbal request? - Did the physician try to discuss euthanasia with the patient? Did the patient understand the explanation? - In what way was communication with the patient about euthanasia still possible (verbal / non-verbal)? - What did you think about the patient either understanding or not understanding what was going to happen? |
|  | Unbearable suffering, without prospect of improvement | - In your opinion, is/was there unbearable suffering? - In your opinion, is/was the situation one without prospect of improvement? - How did the consultation with the physician about this proceed? Were you and the physician on the same page about it? |
|  | Reasonable alternatives | - Did you discuss other treatment options with the physician? Were these tried? - Why were alternatives in this case not considered reasonable / why were there no alternatives? |
|  | Performance of euthanasia | - Was euthanasia ultimately carried out or not? Did you support this? Did you feel that you were able to contribute to the decision about whether euthanasia would be carried out? - Was the procedure, and the explanation of it, clear and sufficient for you? - How did the performance of euthanasia proceed? - Did the patient show resistance (verbal / physical)? - Did the physician take enough time and approach the procedure calmly? What went well and what could have been improved? |
|  | Premedication | - Did the patient receive premedication? Would the patient have wanted this or not? - Did the physician ask the patient / you for permission for this? - What is your view on giving premedication prior to carrying out euthanasia? |
| Emotional burden | Impact on family members | - What impact did the procedure leading up to euthanasia (and the euthanasia itself) have on you? - Did you experience the euthanasia process, or the consideration of euthanasia, as emotionally difficult? - Were you able to discuss this topic with the physician? |
|  | Impact on physicians | - What impact do you think the procedure had on the physician (and other healthcare professionals)? - Was this discussed? |

** This is an English translation of the original Dutch topic list.*

**Appendix 3 |** Topic Guide Interview with Healthcare Professionals*

| **Topics** | **Sub-topics** | **Example questions** |
| --- | --- | --- |
| Problem outline |  | - How often do you encounter patients with advanced dementia who have a written advance directive? - Does it ever happen that patients or their relatives later decide not to rely on the directive after all? What reasons are given for this? - How often is there a request for euthanasia to be performed? How often is it actually carried out? |
| **Case-specific section** | | |
| Case | Situation outline | - Can you describe the situation in this specific case? |
| Written advance euthanasia directive | Treatment relationship and discussion of the advance euthanasia directive | - How long was the treatment relationship you had with this patient? - How often did you discuss the directive with the patient / the patient’s relatives? - Who was involved? How was the case handed over between healthcare professionals? |
|  | Content of the advance euthanasia directive | - What is the role of the written advance directive? - What did the directive look like? What did it contain? What did you think of its content? - Was the wording clear? Was interpretation possible? |
| Request for euthanasia (if applicable) | Frequency of the request | - At what point did the request for euthanasia arise in this specific case? - Who initiated this request? |
|  | Who was involved | - Who was involved? (family / colleagues / care team, etc.) - Were there any dilemmas within the care team or the family? |
|  | Reason for the request | - What was the patient’s reason for choosing euthanasia? - What was the family’s reason for making the request at this point? - Did you support this reason / were you able to understand it? |
|  | Voluntary and well-considered | - How do you assess the voluntary nature of the request? - Was communication with the patient possible in this case? If so, in what way? - Do you think that in this case the written directive could replace the verbal request? - Whose opinion did you take into account in your decision? |
|  | Unbearable suffering | - How do you assess the unbearable nature of the suffering? How do you assess the hopelessness of the situation? - Whose opinion did you take into account in your decision? |
|  | Reasonable alternative | - Did you consider reasonable alternatives? Which ones? With whom did you discuss them? - Why were these alternatives in this case not considered reasonable? |
| Performance of euthanasia (if applicable) | Performance | - Was euthanasia carried out in this case? Why or why not? - How was this decision reached? How did the performance proceed? |
|  | Duty to inform the patient | - Before the act, was it explained to the patient what actions would be performed and for what purpose? Was the patient able to understand this information? - Do you believe that, as a healthcare professional, you have an ethical duty to discuss this beforehand with the patient? - Do you think it is ethically justifiable to carry out euthanasia if the patient does not understand what is going to happen? |
|  | Resistance during performance | - Was there verbal or physical resistance during the performance? - Would you have performed euthanasia if there had been verbal / physical resistance? |
|  | Premedication | - Did the patient in this case receive premedication? Was anything about this described in the directive? What are your views on premedication? - Do you think that, as a healthcare professional, you have an ethical duty to ask the patient for permission before administering premedication? |
| Dilemmas |  | - Are there any additional dilemmas you encountered in this specific case that have not yet been discussed in this interview? - How did you experience the dilemmas in this case? How did you feel about them? How did you deal with them? |
| **General section** | | |
| Alternative treatment | Other options | - What other treatment options are there, in your view, around the end of life? - Do you think these options can be an alternative to euthanasia? |
|  | Provision of information | - Do you think there is sufficient attention is given alternative treatment options? - Are patients sufficiently aware of the alternative options? |
| Emotional burden | Pressure on physicians | - Do you feel that the pressure has increased? If so, how do you notice this? - To what extent are you influenced by this? |
|  | Impact on physicians | - What emotional burden does euthanasia place on you? What uncertainties do you feel? How do you deal with them? - Do you think the patient and/or the patient’s relatives are sufficiently aware of the impact euthanasia has on the physician? |
| Personal perspective | Perspective on end-of-life care | - What is your view of good end-of-life care for people with dementia? - What is the role of euthanasia in dementia within this? - Is there a difference between your personal perspective and what you consider socially acceptable? - What do you need in your work around euthanasia / end-of-life care? What would that look like? |

** This is an English translation of the original Dutch topic list.*

**Appendix 4 |** Code tree – Euthanasia in advanced dementia

| **A. General** | | | | | |
| --- | --- | --- | --- | --- | --- |
| A1 | Case summary |  |  |  |  |
| A2 | Euthanasia trajectory |  |  |  |  |
| A3 | General information |  |  |  |  |
| A4 | Society (including societal perspective) | A4a | Views on the AED* |  |  |
| A5 | Family experiences (including with healthcare professionals/other family members) |  |  |  |  |
| **B. Decision-making process** | | | | | |
| B6 | Barriers (reasons to forgo) | B6a | Early-stage dementia (voluntary, but no suffering) |  |  |
|  |  | B6b | End-stage dementia (suffering is present, but is the request voluntary?) |  |  |
|  |  | B6c | Questionable AED |  |  |
|  |  | B6d | Different views regarding the euthanasia request (relatives/family) |  |  |
|  |  | B6e | Negative opinion from the SCEN*-physician or another physician/healthcare professional |  |  |
|  |  | B6f | Lack of experience (physician, EEC-team) |  |  |
|  |  | B6g | Short treatment relationship, resulting in insufficient familiarity with the patient |  |  |
|  |  | B6h | Insufficient handover between healthcare professionals (e.g. from GP* to ECP*) |  |  |
| B7 | Facilitators (reasons to comply) | B7a | Long treatment relationship |  |  |
|  |  | B7b | Short treatment relationship, but still sufficient familiarity with the patient |  |  |
|  |  | B7c | Conversations with relatives/family |  |  |
|  |  | B7d | Consultation with colleague physicians or other healthcare professionals, with a positive opinion of the SCEN-physician |  |  |
|  |  | B7e | Good handover between healthcare professionals (e.g. from GP to ECP) |  |  |
|  |  | B7f | Sufficient experience (physician, EEC*-team) |  |  |
| B8 | Personal perspective | B8a | On euthanasia |  |  |
|  |  | B8b | On the euthanasia request |  |  |
|  |  | B8c | On end-of-life care |  |  |
|  |  | B8d | Retrospective discussion of the case or discussion prompted by earlier euthanasia deliberations |  |  |
| B9 | Reason for the decision |  |  |  |  |
| B10 | Personal way of reaching a decision (norms and values related to the case) |  |  |  |  |
| B11 | Needs (e.g. reflection with colleagues, prior review) |  |  |  |  |
| **C. Euthanasia** | | | | | |
| C12 | Euthanasia Act (general) |  |  |  |  |
| C13 | Review committee (including experience with / views on) |  |  |  |  |
| C14 | Professional interpretation (i.e. grey area of ‘mutatis mutandis’) |  |  |  |  |
| C15 | Voluntary and well-considered request | C15a | Capable of making decisions / incapable of making decisions |  |  |
|  |  | C15b | Shifting boundaries |  |  |
|  |  | C15c | AED | C15ci | Importance of the AED for the physician |
|  |  |  |  | C15cii | Adequate / inadequate AED |
|  |  |  |  | C15ciii | Conflict between the AED and the current situation |
| C16 | Unbearable suffering | C16a | Types of suffering (e.g. anticipatory suffering or suffering within the family) |  |  |
|  |  | C16b | Observability/verifiability of suffering (verbal/non-verbal) |  |  |
|  |  | C16c | Personal view of suffering (interpretability/subjectivity/empathic comprehensibility) |  |  |
| C17 | Information and counselling (e.g. advance care planning, preceding trajectory) |  |  |  |  |
| C18 | Reasonable alternative |  |  |  |  |
| C19 | Premedication |  |  |  |  |
| C20 | Performance (how, with whom, and how it proceeded) |  |  |  |  |
| **D. Moral and emotional aspects** | | | | | |
| D21 | Emotional burden | D21a | Healthcare professionals |  |  |
|  |  | D21b | Family |  |  |
| D22 | Moral dilemma (general norms and values) |  |  |  |  |
| D23 | Coping mechanism |  |  |  |  |
| D24 | Pressure |  |  |  |  |

**Legend Appendix 4** | Abbreviations

****AED*** *= advance euthanasia directive;* ***SCEN*** *= Support and Consultation on Euthanasia in the Netherlands;* ***GP*** *= general practitioner;* ***ECP*** *= elderly care physician;* ***EEC*** *= Euthanasia Expertise Center.*

| Available on request. |
| --- |

**Appendix 5 |** Framework matrix

**Appendix 6** | Participants' characteristics and roles in the EAS process

| **Participant number** | **Case involvement** | **Role in EAS process** | **Medical specialization** | **Gender** | **Age** | **Years of working experience** |
| --- | --- | --- | --- | --- | --- | --- |
| **Case 1** – Male, euthanasia performed, AED (+), decisional capacity (+), community-dwelling patient. | | | | | | |
| 1.1a,c | Case-physician | Treating-physician (EEC*-physician) | Internist-hematologist, SCEN^-physician, EEC*-physician | Male | 67 | 41 including 7 as SCEN^-physician and EEC*-physician |
| 1.2e | Stakeholder | EEC*-nurse | Nurse | Female | 65 | 49, including 8 as EEC*-nurse |
| 1.3f | Stakeholder | Patient's child | Not applicable | Female | 60 | Not applicable |
| **Case 2** – Male, euthanasia not performed, AED (+), decisional capacity (+–), nursing home patient. | | | | | | |
| 2.1a,d | Case-physician | Consulting-physician (SCEN^-physician) | Urologist, SCEN^-physician | Male | 53 | 17, including 4 as SCEN^-physician |
| 2.2b,c | Stakeholder | Treating-physician (EEC*-physician) | Internist, SCEN^-physician, EEC*-physician | Male | 67 | 35, including 8 as SCEN^-physician and 6 as EEC*-physician |
| 2.3b,c | Stakeholder | Treating-physician (EEC*-physician) | GP^@^, EEC*-physician | Female | 46 | 14, including 2 as EEC*-physician |
| **Case 3** – Female, euthanasia not performed, AED (+), decisional capacity (+–), community-dwelling patient. | | | | | | |
| 3.1a,c | Case-physician | Treating-physician (GP^@^) | GP^@^, SCEN^-physician | Female | 62 | 32, including 8 as SCEN^-physician |
| 3.2b,d | Stakeholder | Consulting-physician (SCEN^-physician) | ECP^~^, SCEN^-physician | Male | 66 | 32, including 12 as SCEN^-physician |
| **Case 4** – Male, euthanasia performed, AED (+), decisional capacity (+), community-dwelling patient. | | | | | | |
| 4.1a,d | Case-physician | Consulting-physician (SCEN^-physician) | ECP^~^, SCEN^-physician | Female | 70 | 32, including 6 as SCEN^-physician |
| 4.2b,c | Stakeholder | Treating-physician (EEC*-physician) | GP^@^, SCEN^-physician, EEC*-physician | Male | 68 | 30, including 14 as SCEN^-physician and  7 as EEC*-physician |
| 4.3e | Stakeholder | Case manager | Nurse | Female | 62 | 30, including 6 as case manager |
| 4.4f | Stakeholder | Patient's partner | Not applicable | Female | 84 | Not applicable |
| **Case 5** – Male, euthanasia performed, AED (+), decisional capacity (–), nursing home patient. | | | | | | |
| 5.1a,c | Case-physician | Treating-physician (EEC*-physician) | ECP^~^, SCEN^-physician, EEC*-physician | Male | 71 | 30, including 14 as SCEN^-physician and  8 as EEC*-physician |
| 5.2f | Stakeholder | Patient's partner | Not applicable | Female | 70 | Not applicable |
| **Case 6** – Female, euthanasia not performed, AED (+), decisional capacity (–), community-dwelling patient. | | | | | | |
| 6.1a,d | Case-physician | Consulting-physician (SCEN^-physician) | GP^@^, SCEN^-physician | Male | 67 | 40, including 15 as SCEN^-physician |
| 6.2f | Stakeholder | Patient's partner | Not applicable | Male | 70 | Not applicable |
| **Case 7** – Female, euthanasia not performed, AED (+–), decisional capacity (–), nursing home patient. | | | | | | |
| 7.1a,c | Case-physician | Treating-physician (ECP^~^) | ECP^~^, SCEN^-physician | Male | 60 | 24, including 7 as SCEN^-physician |
| **Case 8** – Female, euthanasia performed, AED (+), decisional capacity (–), nursing home patient. | | | | | | |
| 8.1a,c | Case-physician | Treating-physician (ECP^~^) | ECP^~^, SCEN^-physician | Female | 69 | 10, including 8 years as SCEN^-physician |
| **Case 9** – Male, euthanasia performed, AED (+), decisional capacity (+–), community-dwelling patient. | | | | | | |
| 9.1a,d | Case-physician | Consulting-physician (SCEN^-physician) | ECP^~^, SCEN^-physician, GP^@^ | Male | 61 | 11 as ECP, including 5 as SCEN^-physician, 15 as GP^@^ |
| **Case 10** – Male, euthanasia not performed, AED (+–), decisional capacity (–), nursing home patient. | | | | | | |
| 10.1a,c | Case-physician | Treating-physician (EEC*-physician) | Non-specialized physician, EEC*-physician | Female | 69 | 13, including EEC*-physician |
| 10.2e | Stakeholder | EEC*-nurse | Nurse | Female | 60 | 43, including 5 as EEC*-nurse |
| 10.3b,d | Stakeholder | Consulting-physician (ECP^~^) | ECP^~^ | Female | 38 | 12 |
| 10.4b,d | Stakeholder | Consulting-physician (GP^@^) | GP^@^ | Male | 65 | 36 |
| 10.5f | Stakeholder | Patient's partner | Not applicable | Female | 61 | Not applicable |
| **Case 11** – Male, euthanasia performed, AED (+), decisional capacity (+–), nursing home patient. | | | | | | |
| 11.1a,c | Case-physician | Treating-physician (ECP^~^) | ECP^~^ | Female | 64 | 35 |
| **Case 12** – Male, euthanasia performed, AED (+), decisional capacity (+), nursing home patient. | | | | | | |
| 12.1a,d | Case-physician | Consulting-physician (SCEN^-physician) | Pediatric neurologist, SCEN^-physician | Female | 71 | 40 , including 4 as SCEN^-physician |
| 12.2b,c | Stakeholder | Treating-physician (EEC*-physician) | GP^@^, SCEN^-physician, EEC*-physician | Female | 70 | 34, including 19 as SCEN^-physician and  8 as EEC*-physician |
| **Case 13** – Female, euthanasia not performed, AED (+), decisional capacity (–), nursing home patient. | | | | | | |
| 13.1a,c | Case-physician | Treating-physician (ECP^~^ in training) | ECP^~^ | Female | 35 | 9 |
| **Case 14** – Male, euthanasia not performed, AED (+), decisional capacity (–), nursing home patient. | | | | | | |
| 14.1 | Case-physician | Treating-physician (ECP^~^) | ECP^~^ | Female | 43 | 10 |
| *a: case-physician, b: stakeholder-physician, c: treating-physician, d: consulting-physician, e: other healthcare professional, f: relative, +: yes,* –*: no, +*–*: doubts, *EEC: ‘Euthanasia Expertise Center’, ^SCEN: ‘Support and Consultation on Euthanasia in the Netherlands’, ^@^GP: general practitioner, ^~^ECP: elderly care physician.* | | | | | | |


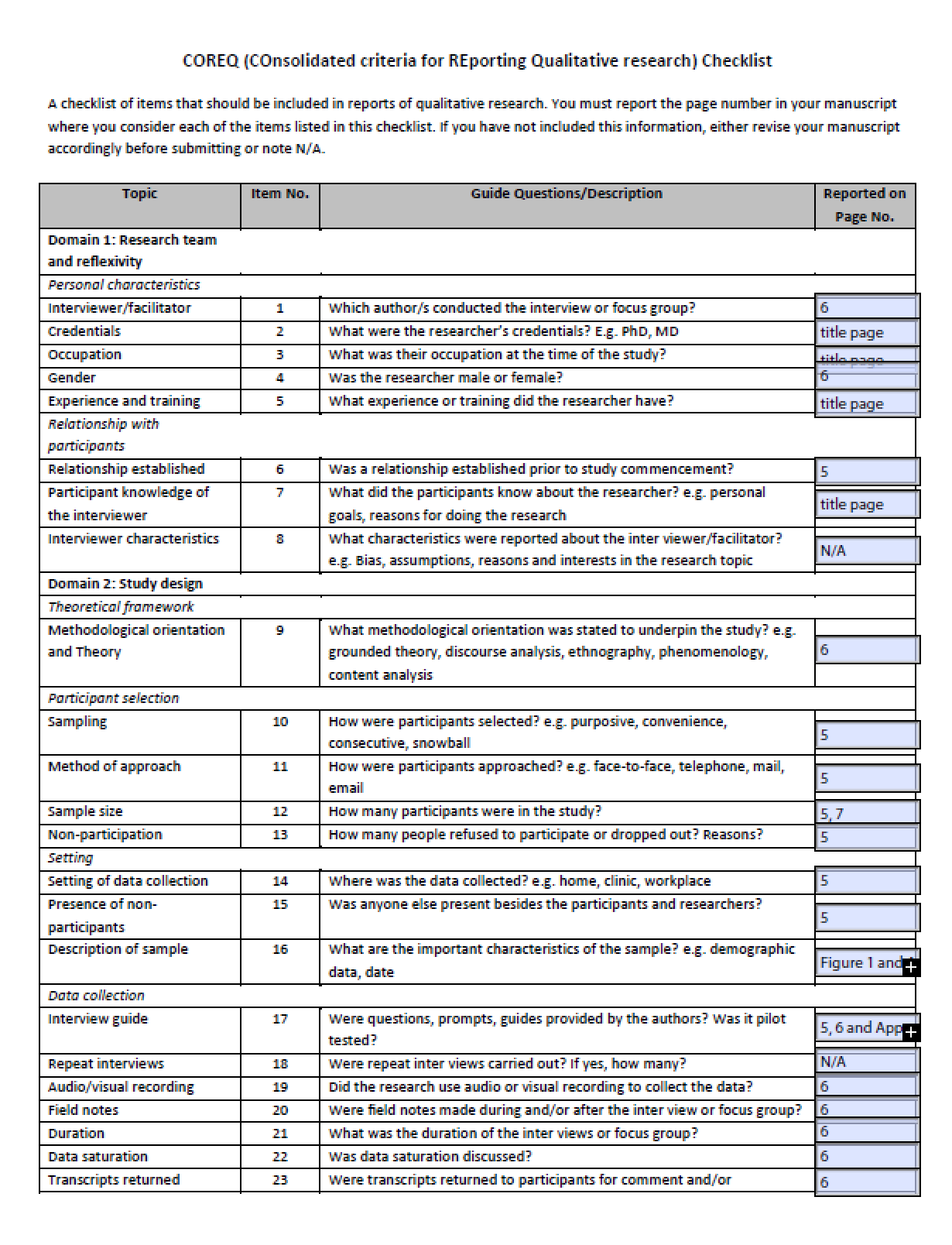
**Appendix 7** | COREQ checklist


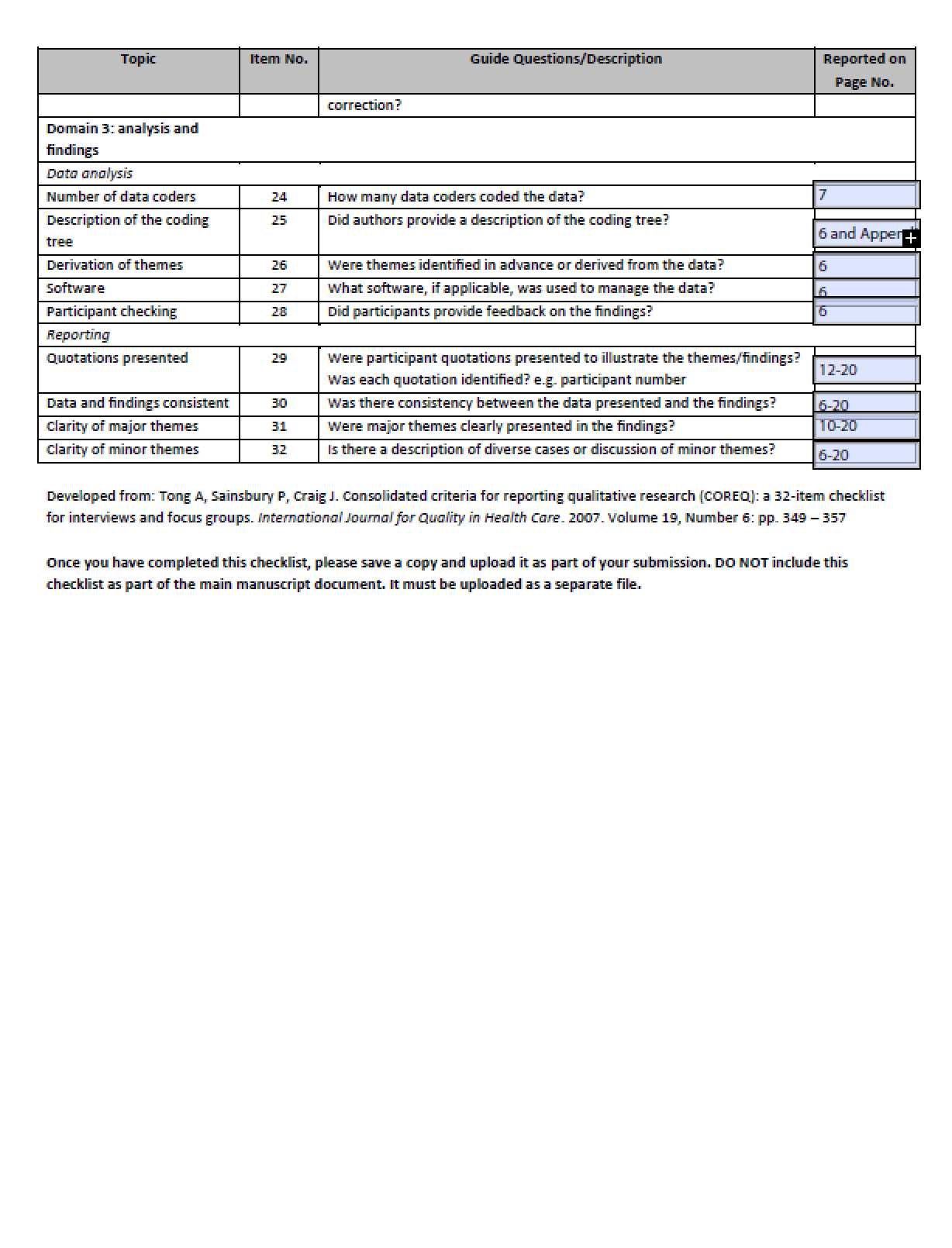

Supplement: Supplementary_materials_afag170 [file supplementary_materials_afag170.zip › Supplementary_materials_afag170.docx]
